# Every-Other-Day Feeding Prevents the Loss of Parvalbumin-Expressing Neurons in the Cerebral Cortex of Female 5xFAD Mice

**DOI:** 10.1007/s12035-025-05355-w

**Published:** 2025-11-28

**Authors:** Jelena Ciric, Milka Perovic, Nikola Milovanovic, Suryanarayana Polaka, Irena Jovanovic Macura, Natasa Nestorovic, Vesna Tesic

**Affiliations:** 1 Laboratory for Molecular Neurobiology and Behavior, Department of Neurobiology, Institute for Biological Research “Sinisa Stankovic” - National Institute of the Republic of Serbia, University of Belgrade, Belgrade, Serbia; 2 Department of Neurology, Louisiana State University Health Shreveport, Shreveport, LA USA; 3 Department of Pathology, Louisiana State University Health Shreveport, Shreveport, LA USA; 4 Department of Cytology, Institute for Biological Research “Sinisa Stankovic” - National Institute of the Republic of Serbia, University of Belgrade, Belgrade, Serbia; 5 Institute for Cerebrovascular and Neuroregeneration Research (ICNR), Louisiana State University Health Shreveport, Shreveport, USA

**Keywords:** Alzheimer’s disease, Food restriction, EOD feeding, Parvalbumin, BDNF, CBP

## Abstract

The present study demonstrates the effects of an every-other-day (EOD) feeding regimen on parvalbumin (PV)-expressing interneurons in the cortex of 5xFAD mice, a well-established animal model of Alzheimer’s disease (AD). Female 5xFAD mice and their non-transgenic littermates were maintained on either an ad libitum (AL) or EOD feeding regimen throughout the presymptomatic phase of the pathology, with comprehensive immunohistochemical and Western blot analyses performed in 6-month-old animals. In AL-fed 5xFAD mice, significant reductions in PV-expressing interneurons were observed in the retrosplenial granular, parietal, and somatosensory cortices compared to non-transgenic controls, supporting their established vulnerability in AD pathology. This neuronal loss was accompanied by a decline in the levels of brain-derived neurotrophic factor (BDNF), a key neurotrophin essential for cell survival and synaptic plasticity. Remarkably, four months of EOD feeding prevented the Aβ-induced loss of PV interneurons and increased the total protein levels of the BDNF receptor TrkB, suggesting enhanced neurotrophic signaling. However, the benefits of EOD feeding were not uniform across all molecular markers, with EOD-fed 5xFAD mice retaining deficits in phosphorylated CaMKII and CREB-binding protein (CBP) and biochemical analysis of plasma indicating metabolic stress-related effects. Together, these results align with the GABAergic hypofunction hypothesis in AD, underscoring the importance of PV interneuron plasticity in neurodegeneration and cognitive decline. They also suggest that dietary strategies like EOD feeding may offer partial neuroprotective effects during early AD progression, however, complex stress-related impacts of EOD in the modulation of PV neuron function remain to be elucidated. Future studies are also warranted to more carefully explore the long-term translational potential of dietary interventions and the interplay between metabolic stress and amyloid pathology, in order to identify novel therapeutic targets across distinct neuronal populations.

**Supplementary Information:**

The online version contains supplementary material available at 10.1007/s12035-025-05355-w.

## Introduction

Alzheimer’s disease (AD) is the most prevalent age-associated brain disorder, clinically characterized by progressive cognitive decline and behavioral deficits [1]. Brain accumulation of amyloid-β (Aβ) in senile plaques and hyperphosphorylated tau (pTau) protein in neurofibrillary tangles has a causal role in AD pathogenesis that leads to synaptic and neuronal loss primarily in the cortex and hippocampal formation [2]. In addition, the disease is characterized by a decades-long, clinically silent prodromal phase, underscoring the urgent need for novel treatment strategies focused on prevention [3].

Profound remodeling and dysfunction of the GABAergic system, which is crucial for maintaining the balance between excitatory and inhibitory (E/I) neural activity, have been reported in both patients and various mouse AD models [4]. Epidemiological studies consistently demonstrated an eightfold higher incidence of seizures in individuals with AD than people without dementia [5], and with early-onset AD patients being more prone to seizures or epilepsy than those with a late-onset AD [6, 7]. Neural network dysfunction due to reduced GABA levels and loss of GABAergic interneurons was further linked to a more rapid decline in cognitive domains such as complex attention, executive function, and learning and memory [8, 9]. Aberrant network activity was also reported as an early event in various mouse AD models [10–12]. Moreover, treatment with the antiepileptic drug levetiracetam (LEV) has been shown to reverse both synaptic deficits and cognitive impairments in different lines of transgenic mice [13]. In people with amnestic mild cognitive impairment, LEV also managed to reduce cognitive deficits [14]. Neural network hyperactivity is thus an emerging functional hallmark of AD, contributing to pathogenesis and associated cognitive decline.

Recently, a significant step forward has been achieved by linking Aβ-induced neuronal network dysfunction to a subset of GABAergic interneurons expressing the calcium-buffering protein parvalbumin (PV) [11, 15]. By binding calcium with high affinity, PV protects mitochondria from Ca^2+^ overload and thus has an important role in presynaptic calcium dynamic signaling and synaptic integration [16, 17]. PV-containing interneurons (PV-INs) are widely distributed throughout cortical and hippocampal circuits and are characterized by their rapid, non-adaptive firing patterns, which is why they are commonly referred to as fast-spiking interneurons [18]. By targeting the soma or axon initial segments of excitatory principal neurons, PV-INs regulate local network dynamics, including theta and gamma oscillations critical for memory encoding and retrieval [15, 19, 20]. Chandelier and basket PV-INs constitute up to 50% of inhibitory interneurons in human prefrontal cortical regions. Similarly, in the cortex of mice, PV-INs represent the largest interneuron population (40–50% of all interneurons), followed by somatostatin- and calbindin-expressing interneurons, which account for approximately 20–30%, and 10–20%, respectively [21]. Together, these major interneuron subtypes exert distinct and complementary roles in modulating cortical circuit activity, synchronizing neuronal networks, and maintaining E/I the balance. Furthermore, functional heterogeneity among PV-INs subpopulations has even been defined based on distinct schedules of neurogenesis, input connectivity, output target neurons, and roles in learning [22]. Prefrontal PV-INs were demonstrated to govern short-term memory and social behavior in mice [23, 24].

In addition, PV-INs were demonstrated to be highly sensitive to oxidative stress [25, 26]. Post-mortem immunohistochemical analysis confirmed a marked and region-selective PV-INs loss in the entorhinal cortex and DG and CA1-CA2 subfields of the hippocampus of AD patients [27, 28]. In some studies, however, no obvious decline was observed in the temporal cortex, perirhinal cortex, CA3, subiculum, and presubiculum [29, 30]. Paradoxically, PV-IN density was found to be increased in the piriform cortex, suggesting compensatory mechanisms [31]. In mouse AD models, more consistent PV-IN loss was observed, with PV-IN density declining by 40–50% in CA1 and DG regions of the hippocampus, and correlating with the severity of Aβ deposition [32–34]. Higher AD pathology was also associated with a lower density of PV-expressing interneurons in humans [35]. However, limited data related to PV-INs in the cortex exist.

Early interventions aimed at restoring PV-IN activity were also proven to have long-term beneficial effects on memory and network activity and to reduce amyloid plaque deposition [11, 36–38]. Alternative approaches, such as neuromodulation, also suggested a novel strategy for reversing memory deficits in recent optogenetic or chemogenetic studies [39]. Food restriction (FR) is a widely accepted non-genetic and non-pharmacological approach that extends lifespan, slows down physiological aging, and delays the onset or reduces the severity of age-associated diseases [40, 41]. Neuroprotective effects of FR were also demonstrated in several animal models of epileptic seizures, stroke, and traumatic brain injury [42–45]. Furthermore, we have previously shown that an intermittent, every-other-day (EOD) feeding regimen can counteract age-related changes in glucocorticoid signaling and the expression of presynaptic proteins [46, 47]. In the 5xFAD mouse model of AD, we found, however, that preventive EOD feeding increased inflammation without affecting Aβ load or blood–brain barrier permeability, suggesting that caution should be taken when using food restrictions in the presymptomatic phase of the pathology [48].

5xFAD mice co-overexpress five human familial AD gene mutations and recapitulate many pathological features of AD, including inflammation and progressive neuronal death [49, 50]. Given that two-thirds of AD patients are women, who also exhibit a higher progression rate and greater disease severity than men, but also having in mind more pronounced pathology and response to treatments observed in females of this particular AD mouse model [51, 52], we used female 5xFAD mice in the present study. The EOD regimen was applied during the entire presymptomatic phase of the pathology, and the effects of the EOD feeding regimen on PV interneurons and brain-derived neurotrophic factors (BDNF)/TrkB signaling were evaluated in the cortex during the early symptomatic phase. Activity-dependent changes in gene expression, i.e., excitation-transcription (E/T) coupling, were also assessed by the levels of calcium/calmodulin kinase II (CaMKII)-dependent phosphorylation mediating cyclic AMP-responsive element-binding protein (CREB) activation. Furthermore, the CREB-binding protein (CBP), which facilitates the recruitment of RNA polymerase II to gene promoters and is essential for synaptic plasticity, was evaluated [53]. The same pool of animals from the prior study was analyzed, with the addition of EOD-fed non-transgenic mice, to ensure comparability and direct correlation of the present findings with the broader set of parameters reported previously. The main study aim is thus to further elucidate the effects associated with the EOD feeding regimen on PV-INs in the presence of AD pathology. The focus on PV-INs as a vulnerable yet potentially adaptive neuronal population in AD was also aimed at better understanding their potential contribution to disease progression and/or neuroprotection.

## Methods

### Animals and Feeding Regimens

A total of 36 female mice was used in the study. 5xFAD transgenic mice (Tg; n = 18), and their non-transgenic littermate controls (non-Tg; n = 18) were F2-offspring of hemizygous 5xFAD transgenic F1 mice obtained by crossing homozygous transgenic male 5xFAD mice purchased from the Jackson Laboratory with B6SJLF1/J female mice (Strain #: 3484-JAX and 100012-JAX, respectively, Bar Harbor, Maine, USA). After weaning (postnatal day 21, P21), genotyping was performed by PCR using tail DNA and the standard protocol recommended by the manufacturer. 5xFAD mice carry five familial AD mutations: Swedish (K670N, M671L), Florida (I716V), and London (V717I) mutations in human amyloid precursor protein (APP695) and two mutations (M146L and L286V) in the human presenilin 1 protein under the control of the neuron-specific Thy-1 promoter [49]. Amyloid β deposits and neuroinflammation can be observed in the cortex and hippocampus as early as 2 months of age, while behavioral deficits can be detected starting at the age of 4–6 months [49, 54]. One of the main advantages of this model is the robust age-related neuronal loss in regions such as the subiculum, cortical layer V, and the medial septum, features notably not observed in other AD transgenic models [50].

All animal procedures were in compliance with Directive 2010/63/EU on the protection of animals used for experimental and other scientific purposes, and were approved by the Ethical Committee for the Use of Laboratory Animals of the Institute for Biological Research "Siniša Stanković", University of Belgrade. (N^o^ 03–03/19). The minimal necessary number of animals was used according to G power analysis software (*n* = 9), and all efforts were made to minimize animal suffering in line with the Guideline on the principles of regulatory acceptance of 3Rs (replacement, reduction, refinement) testing approaches, European Medicines Agency (EMA/CHMP/CVMP/JEG-3Rs/450091/2012).

The animals were housed under standard conditions (*n* = 5 per cage; 23 ± 2 °C, 60–70% relative humidity, 12-h light/dark cycle) with standard laboratory chow (Veterinarski zavod Subotica, Serbia) and water available ad libitum (AL). At the age of 2 months, animals were randomly divided into the AL group that continued to receive food ad libitum, whereas the group on food restriction was fed ad libitum every other day (EOD). For EOD-fed animals, food was placed shortly before the onset of the dark cycle and removed after 24 h, with this type of feeding regimen followed for 4 months (*n* = 9 per group). The general health of the animals was routinely checked, while the body weight was measured every other week.

### Marble-Burying Test

To assess anxiety-like, compulsive-like, or repetitive behaviors, mice were subjected to behavioral testing in the marble-burying paradigm (MBT) at the age of 6 months. The test was conducted during the light cycle between 9:00 a.m. and 12:00 p.m. Mice from the EOD group were tested on the feeding day. In brief, 12 identical glass marbles (*d* = 1 cm) were evenly distributed (three marbles per four rows) over the 5 cm-thick sawdust in the novel standard home-cage. Mice were individually placed in the corner and left undisturbed to explore the cage for 30 min. After 30 min, each animal was removed from the box, and the number of marbles buried was counted. A marble was classified as buried if at least two-thirds were covered by sawdust. In addition, the latency time to the first digging was measured. Following the testing of each animal, the sawdust was changed, and the marbles were cleaned with a 10% ethanol solution and dried with a towel.

### Tissue Collection

After overnight fasting, 6-month-old mice were anesthetized by intraperitoneal injection of ketamine (100 mg/kg) and xylazine (10 mg/kg). Blood was collected by cardiac puncture, followed by transcardial perfusion with ice-cold 0.1 M phosphate-buffered saline (PBS, pH 7.4) for subsequent brain collection. The brains were quickly removed from the skull and divided into halves on ice. From the right hemisphere, cortices were dissected, snap-frozen in liquid nitrogen, and stored at −80 °C for Western blot (WB) analyses. The left hemisphere was fixed in fresh 4% paraformaldehyde/PBS for 24 h and cryoprotected in graded sucrose solutions in PBS (10%−30% w/v) until saturation. Coronal brain sections (30 µm thick) were cut rostro-caudally, using a cryostat (Leica, Wetzlar, Germany), and free-floating sections were stored at −20 °C in a cryoprotective buffer (0.05 M phosphate buffer, 25% glycerol, and 25% ethylene glycol). Sections were collected from −1.656 to −2.255 mm from the bregma according to The Allan Mouse Brain Atlas (2008; Allen Institute for Brain Science, Allen Mouse Brain Atlas, http://mouse.brain-map.org/static/atlas). The serum was isolated and frozen.

### Serum Analysis

All measurements were carried out with commercially available kits (Instrumentation Laboratory, Milan, Italy). Reagent solutions were prepared in accordance with the manufacturer’s instructions, and the assays were run on an ILab 1800 clinical chemistry analyzer (Instrumentation Laboratory, Milan, Italy). Serum glucose concentrations were determined by the glucose oxidase method, whereas cholesterol and triglycerides were analyzed using enzymatic methods.

### Immunohistochemistry

Brain sections were rinsed 3 times for 5 min with 0.1 M PBS, pH 7.4. To avoid nonspecific background staining due to endogenous peroxidase, sections were incubated in 3% H_2_O_2_, 10% methanol in PBS for 15 min at RT. Vectastain Universal Elite ABC kit (Vectastain, PK-6200) was used as an avidin/biotin-based peroxidase system, according to the manufacturer’s instructions. Following 1 h at RT in blocking solution, sections were incubated overnight at 4 °C with monoclonal anti-PV antibody (clone PARV-19; Sigma, P3088; 1:2,000) in 3% NGS/0.1% Triton X-100/PBS. The sections were next incubated in DAB (Vector, SK-4100) for 5 min and rinsed for 5 min in tap water, distilled water, and PBS, in that order. Sections were mounted on the slides, dehydrated, and coverslips were placed using Permount™ Mounting Medium (Fisher Chemical). To confirm immunolabeling specificity, a control experiment was performed in which the primary antibody was omitted.

### Light-Microscope Imaging and Quantifications of PV-Positive Interneurons

Quantitative analysis of PV-immunoreactive interneurons was performed on images taken by light microscopy at magnification ×10, using AxioObserver Microscope Z1 and AxioVision 4.6 software system (Carl Zeiss). The digital images were saved in TIFF format, and representative images were obtained from 15–20 overlapping images using the Image Composite Editor software (Microsoft). Finally, images were exported to *ImageJ*, version 1.74 (NIH; v1.46; http://imagej.nih.gov/ij).

For each animal, 4 sections 150 µm apart were used to ensure systematic sampling principles and provide quantitative rostro-caudal analysis. The total number of PV-positive interneurons was further delineated in four cortical regions—retrosplenial granular cortex (RSG), retrosplenial dysgranular cortex (RSD), parietal cortex (PtA), and somatosensory cortex (S). Regional separation and analysis were guided by a mouse brain atlas and key histological landmarks. Regions of interests were drawn using the freehand selection tool in ImageJ (schematic representation in Fig. 2A), and the total PV^+^ number was counted by the experimenter blinded to genotype and feeding regimen applied, using *ImageJ*. Cells were considered positive according to the previously published criteria [55]. The number of all detected neurons in a given brain region was averaged to yield one final value per animal for that region.

### Western Blot

Cortical tissue was homogenized and sonicated on ice (3 × 5 s at 10 MHz, Hielscher Ultrasound Processor) in 10 volumes (w/v) of RIPA buffer (50 mM Tris–HCl, pH 7.5, 150 mM NaCl, 1% NP-40, 0.1% SDS, 0.5% Triton X-100, 1 mM EDTA, 1 mM EGTA) with protease and phosphatase inhibitors (Roche Diagnostics, Basel, Switzerland). The supernatants were collected following centrifugation at 20,000xg at 4 °C for 30 min, and protein concentration was determined using the Micro BCA Protein Assay Kit (Pierce Biotechnology, Massachusetts, USA). Equal amounts of proteins (10 or 15 μg per lane) were separated by SDS–polyacrylamide gel electrophoresis (10 or 12%) and blotted onto PVDF membranes (Immobilon-P, Merck, USA). Following the incubation for 1 h at room temperature (RT) in 5% non-fat dry milk/TBST (150 mM NaCl, 50 mM Tris, pH 7.4, and 0.1% Tween-20), membranes were incubated overnight at 4 °C in the following primary antibodies with 2% non-fat dry milk: rabbit anti-BDNF (N-20, Santa Cruz Biotechnology, sc-546; 1:500), rabbit anti-pro-BDNF (Merck, AB5613; 1:3,000), rabbit anti-TrkB (794, Santa Cruz Biotechnology, sc-12; 1:7,500), rabbit anti-pTrkB (Tyr^816^, Abcam, ab75173; 1:70,000), rabbit anti-p-CaMKIIα (Thr^286^, Santa Cruz Biotechnology, sc-12886; 1:50,000), rabbit anti-CBP (Santa Cruz Biotechnology, sc-369; 1:300). Appropriate Horse Radish Peroxidase (HRP)-conjugated secondary antibodies were next applied (Santa Cruz Biotechnology; 1:2–5,000) and HRP-immunoreactive bands were visualized using enhanced chemiluminescence (ECL, GE Healthcare, Little Chalfont, UK) and film exposure (Kodak Biomax). Each blot was subsequently re-probed with goat anti-glyceraldehyde 3-phosphate dehydrogenase (GAPDH) (Santa Cruz Biotechnology, sc-20357; 1:1,000) or mouse anti-β-actin antibody (Sigma-Aldrich, A5316; 1:5,000,000) as loading controls. Signals were quantified densitometrically using iBright Analysis Software (Thermo Fisher Scientific, USA) and expressed as relative values, normalized to the corresponding signals for housekeeping genes. The expression of the target proteins in each experimental group was determined relative to the appropriate control value obtained for non-Tg-AL mice that were assigned the value of 100%.

### Statistical Analysis

All values are presented as mean ± SD. Statistical analyses were performed using Statistica 6.0 software (StatSoft Inc., Tulsa, OK, USA). The normality of data sets was assessed using the Shapiro–Wilk test. Where required, data transformations were applied. Group comparisons were carried out by two-way ANOVA, with genotype and feeding regimen as independent factors. Non-Tg-AL animals were used as the reference group for assessing genotype effects, whereas AL-fed 5xFAD mice served as the reference group for EOD-induced effects. Statistical significance was set at *p* < 0.05.

## Results

### EOD Feeding did not Affect Body Weight and Serum Lipid, Glucose, and Liver Enzyme Panel in 5xFAD Mice

**Fig. 1:**
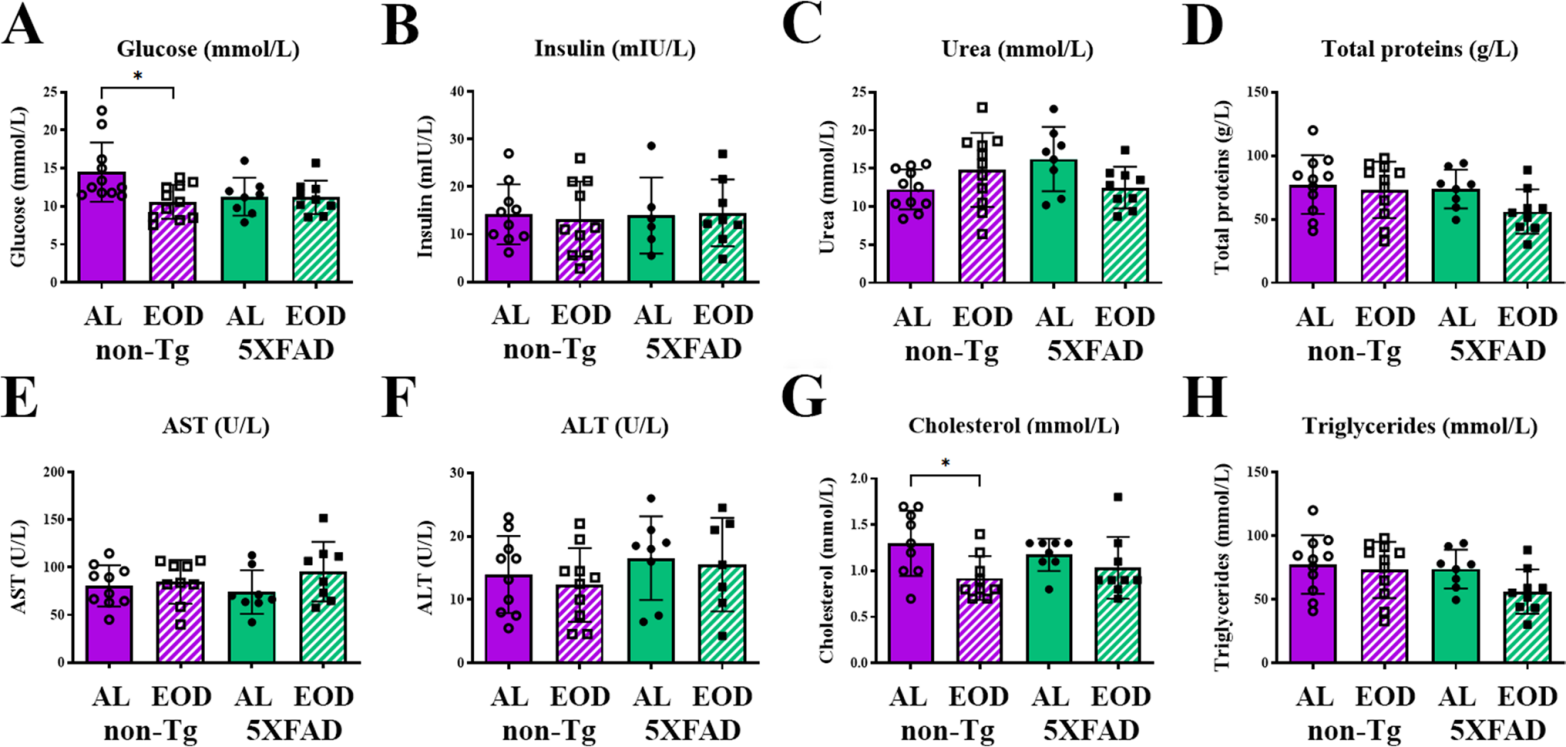
EOD feeding did not affect body weight and serum lipid, glucose, and liver enzyme panel in 5xFAD mice. Body weight **A** and serum levels of glucose **B**, insulin **C**, total proteins **D**, alanine transaminase **E**, aspartate transaminase **F**, cholesterol **G** and triglycerides **H** measured in non-Tg-AL, non-Tg-EOD, Tg-AL, and Tg-EOD mice (*n* = 7–9 mice per group). Data are presented as mean ± SD. *p < 0.05 *vs.* non-Tg-AL mice, as determined by two-way ANOVA

Two-way ANOVA revealed that body weight was not significantly affected by the feeding regimen, irrespective of genotype (Fig. 1A). All animals remained normoglycemic and normocholesterolemic. A significant reduction in glucose (for 27%) was observed only in non-Tg-EOD mice compared with non-Tg-AL controls (Fig. 1B; *p* < 0.05), consistent with the known effects of EOD feeding (F(1, 36) = 5.503, *p* = 0.0246). No genotype-related effects were detected, and all other serum parameters remained unchanged (Figs. 1C–H).

### EOD Feeding Prevented a Reduction in the Number of PV-positive Cells in the Cortex of 5xFAD Mice

**Fig. 2:**
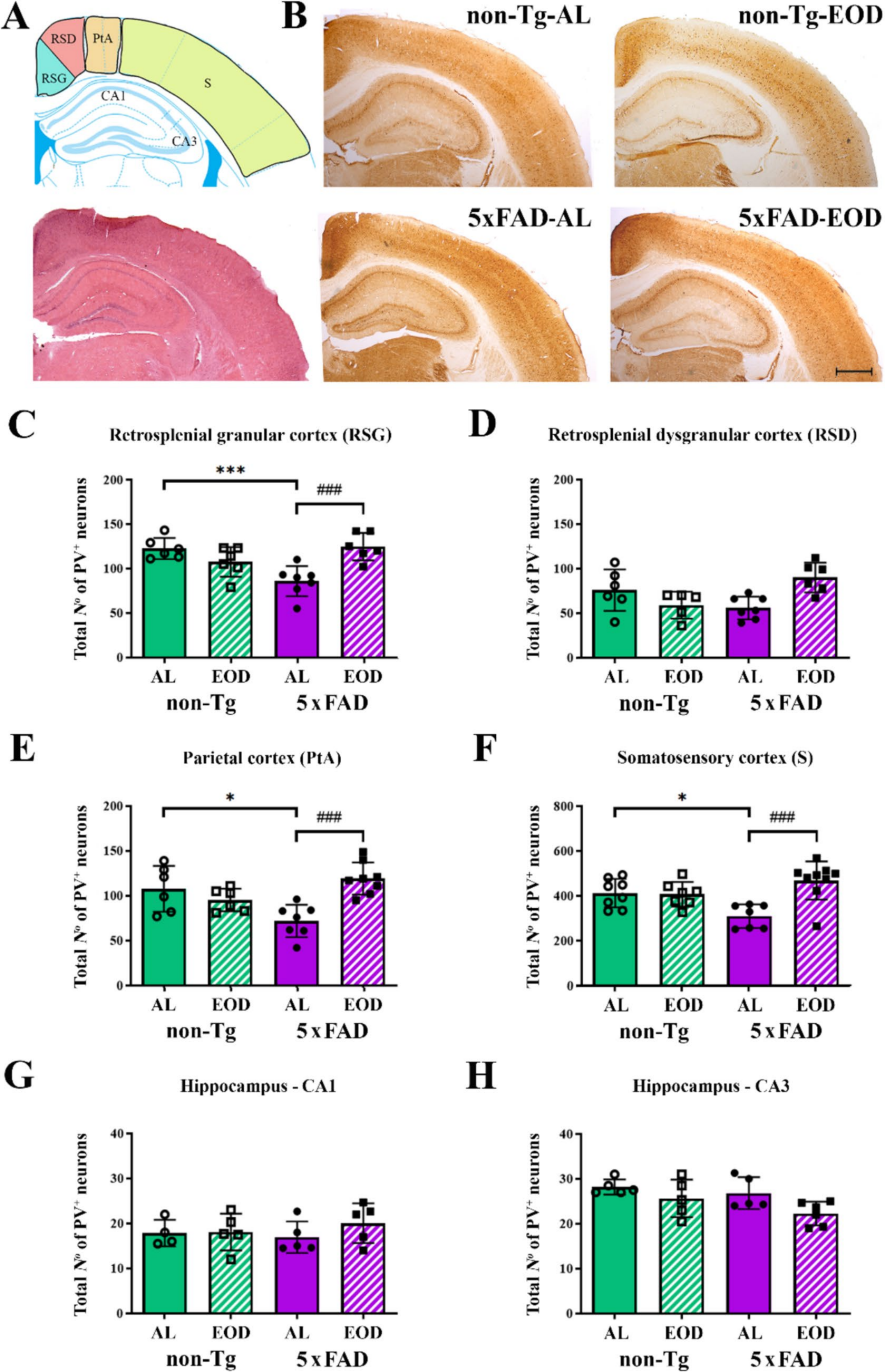
EOD feeding prevents the decrease in PV-INs in 6-month-old 5xFAD female mice. **A** Schematic representation and **B** representative bright field photomicrographs of level-matched parvalbumin (PV)-positive immunostaining in the retrosplenial granular cortex (RSG), retrosplenial dysgranular cortex (RSD), parietal cortex (PtA), somatosensory cortex (S), and hippocampal C1 and CA3 regions. Scale bar = 800 μm. **C–H** Quantitative analysis of the effects of EOD feeding regimen on PV^+^ interneurons in selected cortical and hippocampal regions of non-Tg-AL, non-Tg-EOD, Tg-AL, and Tg-EOD mice (*n* = 9 mice per group). Data are presented as mean ± SD. *p < 0.05 and ***p < 0.001 *vs.* non-Tg-AL mice and ^###^ *p* < 0.001 *vs.* Tg-AL mice, as determined by two-way ANOVA

PV-positive immunoreactivity was observed in the soma, dendritic trees, and axons of stained interneurons (Fig. 2). PV-IN cells were heterogeneous in shape and located mainly within cortical layers 2—6, while in the hippocampus, they were mostly located in the *Stratum pyramidale* and *Stratum oriens* of CA1 and CA3, consistent with previously reported data in the literature.

In the retrosplenial granular cortex, parietal cortex, and somatosensory cortex, a significant effect of feeding regimen (F(1, 17) = 5.006, *p* = 0.0389; F(1, 27) = 3.213, *p* = 0.0843; and F(1, 30) = 7.458, *p* = 0.0105, respectively), as well as the genotype × feeding regimen interaction were observed (F(1, 24) = 27.55, *p* < 0.0001; F(1, 27) = 10.55, *p* = 0.0031; and F(1, 30) = 8.614, *p* = 0.0063, respectively). Post hoc analysis further demonstrated a significant reduction of the number of PV-positive cells in same regions of AL-fed 5xFAD mice in comparison to their non-Tg-AL matched control (Fig. 2). Namely, Tg-AL mice showed a significantly reduced number of PV-positive cells in the retrosplenial granular cortex (for 30.0%; *p* < 0.001; Fig. 2C), parietal cortex (for 30.2%; *p* < 0.001; Fig. 2E), and somatosensory cortex (for 25.1%; *p < 0.05; Fig. 2F), while no changes were detected in the retrosplenial dysgranular cortex (Fig. 2D). Interestingly, four months of EOD feeding reverted the number of PV-positive cells to the control values in all three regions examined (non-Tg-AL vs. Tg-AL group; *p* < 0.001; Figs. 2C, E, F). In the hippocampus, however, no differences in the number of PV-positive neurons in the CA1 and CA3 regions were detected following EOD feeding, either in non-Tg or 5xFAD mice (Figs. 2G, H).

### Impaired Latency in the Marble-Burying Test was Normalized by the EOD Feeding Regimen in 5xFAD Mice

We previously demonstrated that EOD feeding did not affect the mobility and stereotypic behavior in 5xFAD mice in the open field, nor the long-term memory tested within the novel object recognition paradigm [48]. On the other hand, 5xFAD-EOD mice exhibited a significant deterioration in short-term memory and a tendency towards increased anxiety-like behavior in the light/dark box [48]. We next analyzed their behavior in MBT, designed to assess anxiety-like, compulsive-like, or repetitive behaviors in mice [56]. The latency to first digging activity and the total number of unburied, visible were recorded as the measurements of relevant exploratory activity and repetitive-like behavior. High latency to first digging and an increased number of visible marbles indicate anxiety-like or compulsive behavior, respectively.

**Fig. 3:**
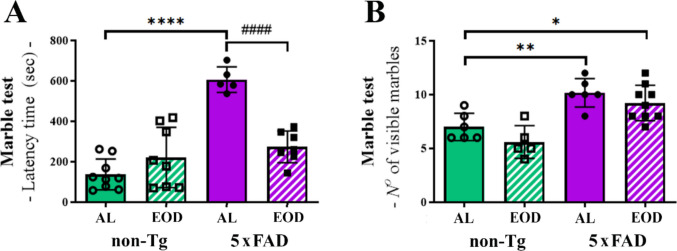
Behavioral performance of EOD-fed 5xFAD mice in the marble-burying test is improved in comparison to AL-fed animals. **A** Latency time and **B** the number of visible marbles for non-Tg-AL, non-Tg-EOD, 5xFAD-AL, and 5xFAD-EOD mice. Data are presented as mean ± SD (*n* = 9 mice per group). *p < 0.05, **p < 0.01 and ****p < 0.0001 vs. non-Tg-AL mice, and ####*p* < 0.0001 vs. Tg-AL, as determined by two-way ANOVA

We found that Tg-AL mice exhibit a significant, approximately sixfold increase in latency time in comparison to non-Tg-AL mice (Fig. 3A; 137,6 ± 76,2 s in non-Tg-AL vs. 606.6 ± 63.4 s in Tg-AL group; *p* < 0.0001). Also, the number of buried marbles was higher in the Tg-AL group of mice when compared with non-Tg-AL mice (Fig. 3B; 1.45 fold, *p* < 0.01). Interestingly, four months of EOD feeding resulted in a significant decrease in latency to digging (237.7 ± 78.9; p < 0.01 vs. Tg-AL group) and no change in the number of marbles buried in the Tg-EOD group (Figs. 3A and B, respectively). Therefore, the interaction effect of genotype and feeding regimen reached statistical significance for latency time (F(1, 25) = 45.90, *p* < 0.0001 and F(1, 25) = 10.47, *p* = 0.0034, respectively), whereas a near-significant trend was evident for the number of buried marbles (F(1, 22) = 33.06, *p* < 0.0001 and F(1, 22) = 3.943, *p* = 0.05970, respectively).

### The Effects of EOD Feeding on BDNF/TrkB Signaling in the Cortex of 5xFAD Mice

**Fig. 4:**
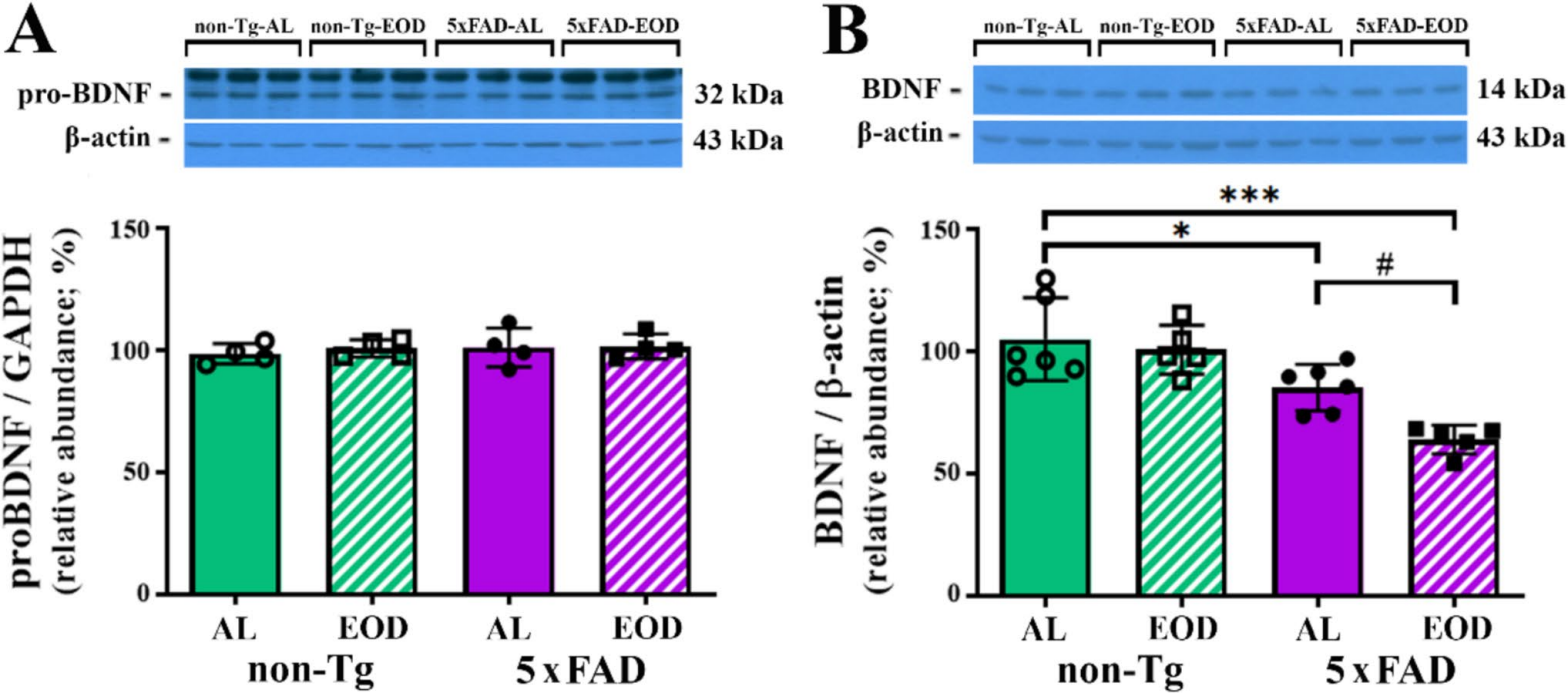
EOD feeding regimen exacerbates the BDNF decrease in the cortex of 6-month-old 5xFAD female mice. Semiquantitative immunoblot analysis of **A** pro-BDNF and **B** mature BDNF protein levels in the cortex of non-Tg-AL, non-Tg-EOD, 5xFAD-AL, and 5xFAD-EOD mice. Protein levels were normalized to the signal of β-actin as a loading control. Representative immunoblots are shown above the graphs. Data are presented as mean ± SD relative to the values obtained in control, non-Tg-AL animals set as 100% (*n* = 7 mice per group). *p < 0.05 and ***p < 0.001 vs. non-Tg-AL mice and #*p* < 0.05 vs. Tg-AL, as determined by two-way ANOVA

BDNF is a well-characterized growth factor in the mammalian brain, playing a critical role in numerous brain functions, including neuronal survival, differentiation, development, and processes such as learning and memory [57]. Impaired BDNF signaling has also been shown to directly influence APP processing and trigger neuronal apoptosis [57]. In contrast, BDNF supplementation reduces the generation of toxic Aβ [58] and restores learning and memory deficits [59]. To investigate the relationship between BDNF and the observed changes in PV^+^ neurons in the cortex of EOD-fed 5xFAD animals and behavioral outcomes, a comprehensive analysis of the BDNF protein level was performed in the cortical tissue of female 5xFAD mice (Fig. 4**)**. Measuring both the expression of BDNF precursor form (pro-BDNF) from which mature BDNF (mBDNF) is generated by proteolytic cleavage is crucial because these forms have distinct, and often opposing, effects on neuronal health. While the mature BDNF promotes synaptic plasticity and neuronal survival, proBDNF can trigger neuronal apoptosis and synaptic dysfunction [57]. Despite no statistically significant differences in the level of pro-BDNF in all experimental groups examined (Fig. 4A), WB analysis of the mature BDNF revealed a significant genotype effect, with its decreased levels in the cortex of both transgenic groups compared with controls (Fig. 4B; F(1, 18) = 20.67, *p* = 0.0002). In particular, a significant decrease in BDNF was identified in the AL-fed 5xFAD mice in comparison to their non-Tg-AL littermates (Fig. 4B; 19.37%; *p* < 0.05). BDNF protein level was further decreased in 5xFAD mice on the EOD feeding regime in comparison with Tg-AL animals (Fig. 4B; 32.77%; *p* < 0.05).

**Fig. 5:**
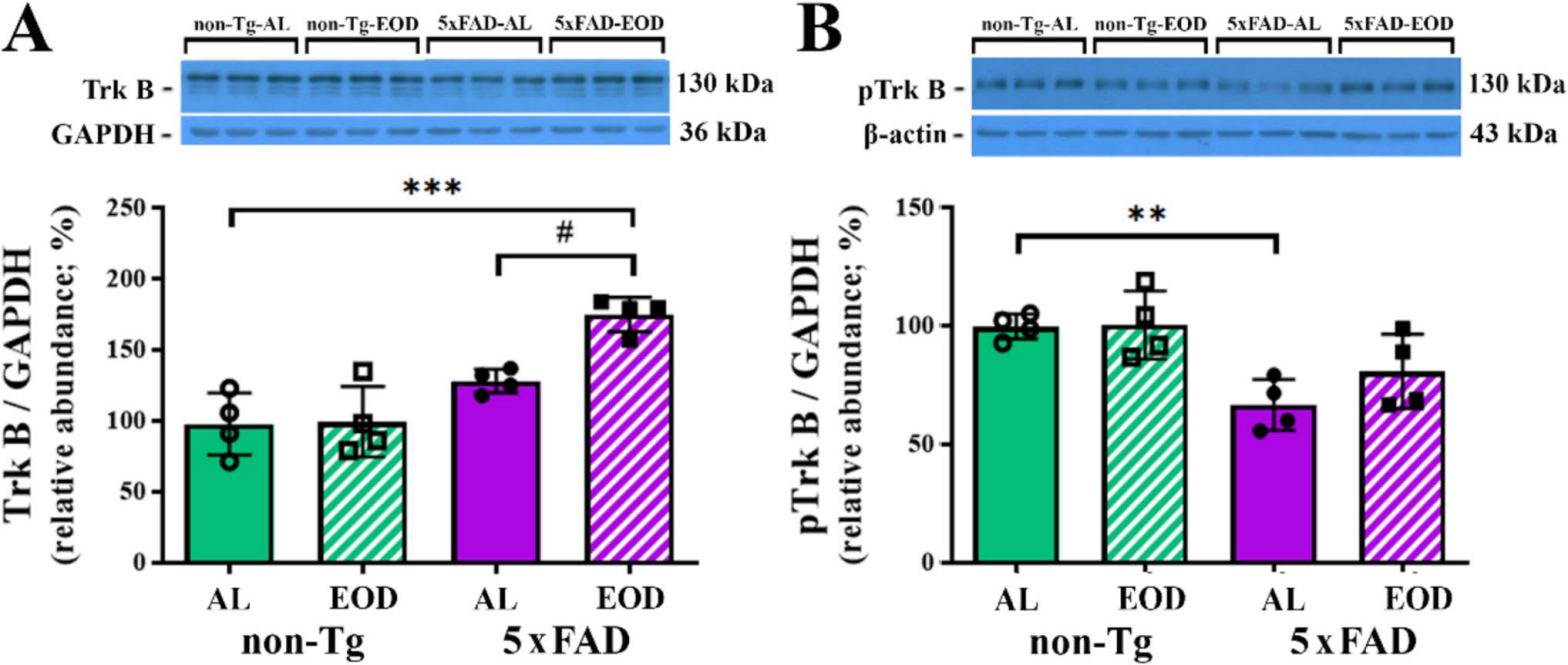
EOD feeding regimen increases TrkB in the cortex of 6-month-old 5xFAD female mice. Semiquantitative immunoblot analysis of **A** TrkB and protein levels of **B** its active form phosphorylated at Tyr^816^ (pTrkB) in the cortex of non-Tg-AL, non-Tg-EOD, 5xFAD-AL, and 5xFAD-EOD mice. Protein levels were normalized to the signal of GAPDH or β-actin as loading controls, respectively. Representative immunoblots are shown above the graphs. Data are presented as mean ± SD relative to the values obtained in control, non-Tg-AL animals set as 100% (*n* = 7 mice per group). **p < 0.01 and ***p < 0.001 vs. non-Tg-AL mice and #*p* < 0.05 vs. Tg-AL, as determined by two-way ANOVA

The biological functions of BDNF are mediated by its binding to a specific cell surface receptor with an intrinsic ligand-sensitive tyrosine kinase activity, TrkB. The binding of BDNF to the TrkB receptor leads to dimerization and autophosphorylation of tyrosine residues in the intracellular domain of the receptor and subsequent activation of cytoplasmic signaling pathways [60]. WB analysis of TrkB, as well as analysis of its active form phosphorylated at Tyr^816^ (pTrkB), revealed changes in the cortex of both 5xFAD groups, Tg-AL, and Tg-EOD-fed mice (Figs. 5A, B, p ≤ 0.045). Namely, two-way ANOVA revealed a significant interaction between genotype and feeding regimen for TrkB expression (*F*(1, 12) = 6.960, *p* < 0.0001), as well as significant main effects of genotype (*F*(1, 12) = 34.08, *p* = 0.0216) and feeding regimen (*F*(1, 12) = 5.225, *p* = 0.0412). Post hoc comparisons indicated, however, that the significant increase in TrkB was confined to the Tg-EOD group, when compared with both the non-Tg-AL and Tg-AL groups (Fig. 5A; p < 0.001 and p < 0.05, respectively). On the other hand, a significant decrease in pTrkB expression was detected only in Tg-AL-fed mice relative to the matched non-Tg-AL control group (for 35%; Fig. 5B; *p* < 0.01), reflecting a robust genotype-induced effect (*F*(1, 12) = 19.17, *p* = 0.0009).

**Fig. 6:**
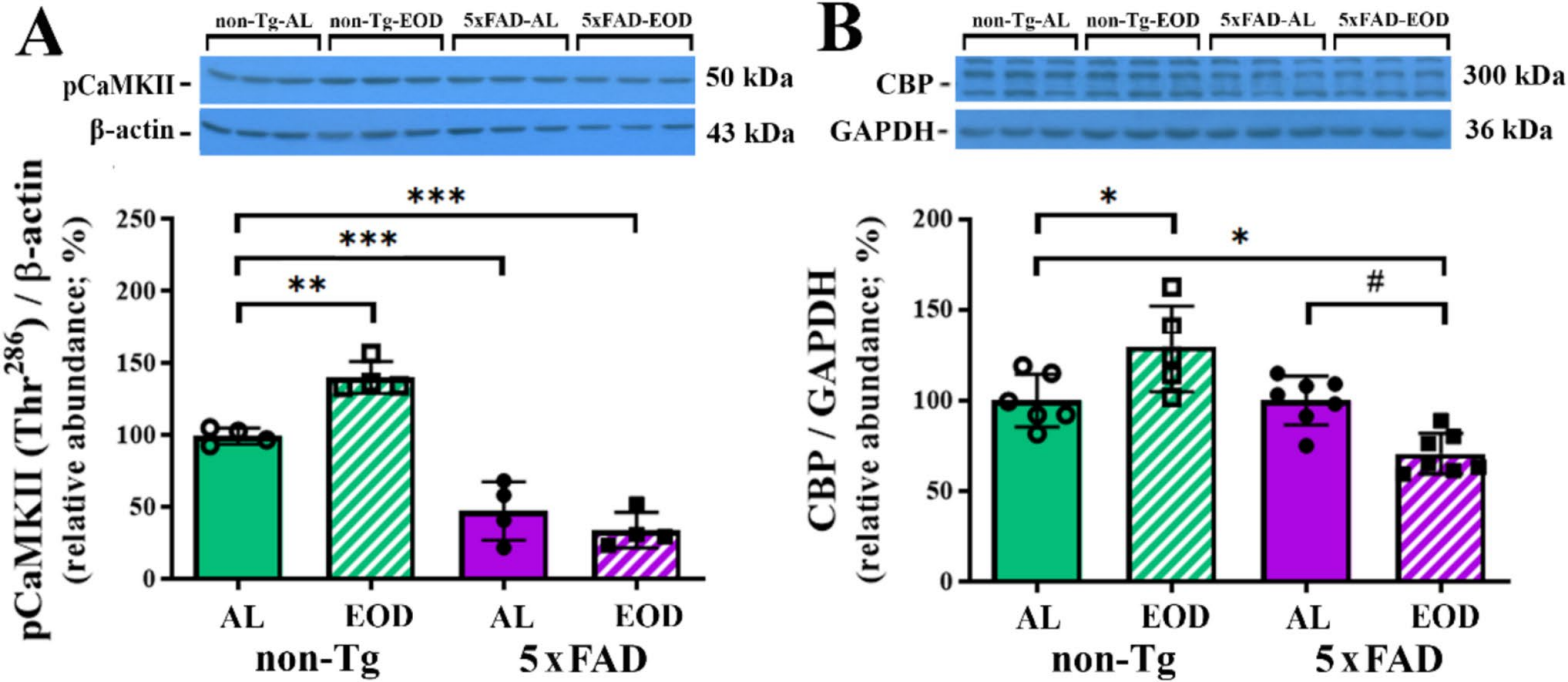
EOD feeding regimen decreases CREB binding protein (CBP) in the cortex of 6-month-old 5xFAD female mice. Semiquantitative immunoblot analysis of **(A)** CaMKII phosphorylated at Thr^286^ (pCaMKII) and **(B)** CBP protein levels in the cortex of non-Tg-AL, non-Tg-EOD, 5xFAD-AL, and 5xFAD-EOD mice. Protein levels were normalized to the signal of β-actin or GAPDH as loading controls, respectively. Representative immunoblots are shown above the graphs. Data are presented as mean ± SD relative to the values obtained in control, non-Tg-AL animals set as 100% (*n* = 7 mice per group). *p < 0.05, **p < 0.01, ***p < 0.001, and ****p < 0.0001 vs. non-Tg-AL mice, respectively, and #*p* < 0.05 vs. Tg-AL, as determined by two-way ANOVA

Calcium/calmodulin-dependent protein kinase II (CaMKII) is a central molecular organizer of synaptic plasticity, learning, and memory, and one of the most important effectors enzymes involved in calcium signaling [61]. Being a Ser/Thr protein kinase, CaMKII is also unique for its autophosphorylation at Thr^286^ (pCaMKII), which is required for high CaMKII activation and initiation of long-lasting synaptic plasticity [62]. In addition, CaMKII mediates BDNF transcription by binding to a calcium response element within the BDNF promoter [63]. Quantitative immunoblot analysis of phosphorylated CaMKII revealed alterations in the cortex of non-Tg mice fed EOD and 5xFAD mice fed both AL and EOD (Fig. 6A). These changes were associated with a highly significant main effect of genotype (*F*(1, 10) = 100.5, *p* < 0.0001). Non-Tg-EOD mice displayed an extensive increase in pCaMKII protein level when compared to non-Tg-AL mice (for 40%; Fig. 6A; *p* < 0.01), while a significant decrease in pCaMKII protein levels was observed in Tg-AL and Tg-EOD mice compared to control mice (Fig. 6A; for 50 and 65%, *p* < 0.001 and *p* < 0.0001, respectively).

We finally examined protein levels of CBP as a coactivator of CREB family transcription factors, which is the main regulator of BDNF gene expression in cortical neurons [64]. Western blot analysis revealed opposite effects of the EOD feeding regimen on the expression of CBP within non-transgenic and transgenic groups. Namely, EOD feeding significantly increased the level of CBP in non-Tg mice in comparison to non-Tg-AL mice by 33.53% (Fig. 6B; *p* < 0.05), whereas EOD feeding significantly decreased the level of CBP in transgenic mice in comparison to Tg-AL mice by 29.4% (Fig. 6B; *p* < 0.05). Statistical analysis further demonstrated a significant interaction between genotype and feeding regimen (*F*(1, 20) = 22.16, *p* = 0.0001).

## Discussion

Although the involvement of parvalbumin-expressing interneurons in AD is well established, the precise mechanisms by which their dysfunction or loss in specific brain regions contributes to disease pathology remain unclear [4]. Furthermore, it is not yet known whether restoring their function can improve functional outcomes [36]. A comprehensive analysis of cortical PV interneurons in AD patients and the transgenic model has also been lacking, and available data are controversial [65]. To address these questions and investigate the GABAergic system as a target for potential clinical investigation in the AD field, we first defined the PV-INs loss in our experimental paradigm. We analyzed next the effects of EOD feeding regimen on the number of PV-INs and BDNF/TrkB signaling pathway in the brain of 5xFAD mice, a well-characterized and widely used transgenic model of AD with robust and early Aβ plaque deposition and concomitant neuroinflammation, particularly in the cortex and hippocampus [49, 50].

The present study demonstrates regional cortical vulnerability in 5xFAD mice, with the loss of PV interneurons detected in most of the cortical regions examined, including retrosplenial granular, parietal, and somatosensory cortex, while no significant effect was detected in the retrosplenial dysgranular cortex. The study also demonstrates that four months of EOD feeding prevents Aβ-induced reduction of PV neurons in these cortical regions. BDNF, a neurotrophin critical for cell survival and neuronal plasticity, was also decreased in the transgenic mice, along with a reduction in the active, phosphorylated form of its receptor, TrkB. However, both proteins were further reduced in EOD-fed animals. Although a compensatory BDNF-induced increase in the total protein level of TrkB was detected with reduced food intake, an increase in pTrkB was absent, consistent with our previous study demonstrating exacerbation in cortical inflammation, downregulation of synaptic plasticity-related proteins, and neuronal injury in EOD-fed animals [48]. No significant difference in pro-BDNF was observed.

Anticipated EOD-induced increase in the protein level of phosphorylated CaMKII and CBP as molecular organizers of synaptic plasticity and learning/memory [61] was, however, confirmed in non-Tg mice. The increase of these proteins in 5xFAD mice thus indicates the well-known vulnerability of cortical PV-INs to Aβ accumulation. Consistently, Aβ oligomers, as the most toxic forms of Aβ that are significantly elevated in 5xFAD mice [49], have been reported to contribute to neuronal death by generating reactive oxygen species, further inducing lipid peroxidation, plasma membrane damage, and an increase in intracellular Ca^2+^ [66]. The pattern of amyloid deposits in the 5xFAD model is considered to closely resemble Aβ pathology in humans, at least in the cortex, with deep layers of frontal cortical regions being particularly vulnerable [33]. Regional and laminar vulnerability was further confirmed by a direct correlation with greater PV neuron loss in the frontal association regions [33, 67]. Although our previous study clearly reported no differences in total Aβ42 levels or Thioflavin S-positive plaque load between AL- and EOD-fed 5xFAD mice in the cortex and hippocampus [48]. In this regard, further studies evaluating soluble Aβ42 or Aβ oligomer levels could be of particular importance.

Our results are also in line with AD-related PV neurodegeneration in humans [27, 28, 68] and previous studies in other AD transgenic mouse models harboring substantial amyloidosis. Reductions in PV-immunoreactive neuron density in the piriform and entorhinal cortices were correlated again with the extent of Aβ deposition [69]. In several other studies, however, no change in PV neuron density was detected in the neocortex [70, 71] or subiculum [72]. Similarly, while in some studies PV-IN loss in the hippocampus has been described [73, 74], no changes were observed in others [75, 76]. The main contribution of our animal study is thus the in-depth analysis of several cortical regions that have not been previously analyzed in detail.

In addition, excessive Aβ was clearly shown to downregulate GABA inhibitory interneuron activity, thereby inducing an E/I imbalance [10–12]. Prolonged upregulation of neural activity was also shown to modulate Aβ peptide secretion by synaptic and non-synaptic Aβ release mechanisms [77] and to increase plaque deposits [78]. Importantly, attenuation of neuronal activity has been associated with reduced axonal dystrophy and synaptic loss around amyloid plaques [37], implicating that its modulation may be a potential therapeutic strategy for AD. The loss of PV-INs might also be an indirect consequence of the loss of other neurons and consequent innervation, including somatostatin-expressing interneurons also reported in 5xFAD mice [50], as recently proposed for neurodegenerative diseases [79].

Parvalbumin neurons are also known to be dominant in areas associated with sensorimotor processing and navigation, such as the retrosplenial cortex (RSC) [80]. In rodents, RSC is a part of the hippocampal-cortical network critical for recent and remote memory retrieval [81, 82]. Although the granular and dysgranular parts of the RSC have shared properties and functions, RSD appears to be more involved in the processing of visual inputs supporting spatial working memory [83]. The lack of EOD-induced effect on visual learning can thus be anticipated, as the granular RSC receives much hippocampal and parahippocampal information, required for the integration of stimuli with a spatial component by RSC excitatory neurons [84]. This regional heterogeneity is also in line with the finding that PV-INs are commonly significantly enriched in proteins associated with cognitive resilience in humans [85]. In 4-month-old 5xFAD mice, RSC was the most affected region with the highest soluble Aβ level [86], and synaptic dysfunction of retrosplenial PV-INs disrupted the ensemble dynamics underlying episodic memory, while their optogenetic activation restored memory retrieval [87]. In the present study, we were, however, unable to detect regional differences in plaque load correlating with the absence of EOD-induced increase in PV-INs, including RSC (**Suppl. Figure 1**).

The EOD feeding during the entire presymptomatic stage in 5xFAD mice aimed to examine possible effects on initiating events contributing to the pathology. Given that early changes in PV-INs could be a causal step in AD-associated network and memory impairment and a significant contributor to disease pathogenesis, they are considered prime targets for intervention before symptom onset [36]. It has also been previously reported that the rapid increase in amyloid pathology occurs in 5xFAD mice until the age of 6 months, which subsequently reaches a certain plateau [49]. Therefore, we cannot exclude that the EOD feeding paradigm led to a certain delay in plaque deposition and consequent neuronal death at earlier time points during pathology progression, which prevented the PV-INs loss we observed by the end of the treatment period. To test this hypothesis, plaque pathology and its relationship to PV-IN function and network rhythmicity can be analyzed at earlier time points. Nevertheless, the overall beneficial effects of the increased number of PV-INs in EOD-fed 5xFAD mice on cognitive deficits are lacking. A partial rescue of behavioral performance in MBT aligns well with anticipated RSD function and implies EOD-induced normalization of the animal’s tendency to explore a novel space despite no amelioration of repetitive behavior. However, this also suggests that the effects of EOD on various aspects of the cognitive and behavioral status and overall health still need further elucidation [48].

With a strong association between dysregulated glucose metabolism and AD pathophysiology [88] and the high energy-demanding physiology of PV-INs [89], mild metabolic stress induced by the EOD feeding regimen may intensify energy restriction within the network and thus exacerbate synaptic dysfunctions. Furthermore, food restriction paradigms are well-known for the moderate elevation of glucocorticoids [90, 91] and the consequent production of stress resistance proteins important for managing stronger stressors [92]. This finding was considered paradoxical, since a prolonged increase in GCs is commonly associated with brain damage [93]. Accordingly, chronic stress was shown to induce pronounced impairments in GABAergic neurotransmission in the prefrontal cortex, particularly in female mice [94–96]. Reduced number of GABAergic neurons, including PV-INs, was also reported [97], revealing the limiting mechanism underlying the control of stress reactivity and behavior. Unpredictable chronic mild stress also reduced glucocorticoid signaling specifically in PV-INs [95], which was morphologically accompanied by interneuron hypertrophy and terminal sprouting [98]. A decrease in PV-IN numbers was observed and further associated with reduced specific protein marker expression rather than stress-induced neurotoxicity. While we did not find EOD-induced alterations in the expression of enzymes necessary for GABA synthesis (glutamic acid decarboxylase (GAD) 65 and GAD67) [48], several other activity-dependent markers of GABAergic transmission [99] may indicate either a functional impairment or a compensatory molecular response. In PV-INs themselves, the downregulation of parvalbumin directly induced an increase in mitochondria volume/density and branching of dendrites, which can be attenuated by PV overexpression [100]. In addition, an increase in dendritic complexity and functional impairments in PV-INs lacking APP suggested that GABAergic interneurons require homeostatic APP levels for proper physiological function and circuit activity control, in contrast to Aβ [101]. Although our previous work demonstrated overall EOD-induced cortical neuritic damage and the increase in the number of axonal spheroids by immunostaining, accompanied by reduced synaptic plasticity markers [48], an important direction for future studies should also include the use of multiple staining approaches combined with sophisticated morphometric analyses, such as assessments of dendritic complexity, to better link regional plaque load, PV interneuron integrity, and their combined contribution to circuit dysfunction in AD.

Multiple other mechanisms could be involved in the higher cortical number of PV-INs following EOD feeding in AD mice. One downstream effect of Aβ overexpression is decreased BDNF levels, which may lead to neuronal and synaptic dysfunction and eventual neurodegeneration [57]. In agreement with previous studies in AD mouse models, we found a positive correlation between decreased levels of BDNF and synaptic/cognitive decline in 5xFAD mice [102]. Recent meta-analysis also reported Aβ-dependent BDNF downregulation in both the hippocampus and cortex of AD patients [103]. Moreover, a consequent decrease in the levels of pCaMKII and pCREB, the activated form of a transcriptional factor that modulates the BDNF expression by recruiting CBP [104], was detected. Our analysis in EOD-fed 5xFAD mice revealed that these proteins related to neuroplasticity and gamma oscillation are not altered in the cortex in the presence of pathology, implying a potential novel underlying mechanism that bypasses the BDNF/Trk signaling pathway. Calpain-mediated cleavage of TrkB receptors, and an increase in mRNA levels of truncated TrkB forms have been shown to be induced by Aβ [105]. Accordingly, the increase in TrkB protein levels observed in EOD-fed 5xFAD mice aligns with the deleterious effects revealed by our previous results [48], including aggravated inflammation, increased neuronal injury and loss, especially in the context of reduced mature BDNF levels and no change in the active form pTrkB.

In conclusion, our findings align with the most recent GABAergic hypofunction hypothesis in AD and further confirm the importance of PV-INs plasticity in neurodegeneration and cognitive impairments [65]. Multiple studies have also demonstrated beneficial anti-aging and neuroprotective effects of food restriction, with specific cellular and molecular mechanisms including the decrease in metabolic rate, increase in insulin sensitivity, protective effects on neurons and synapses, and the increase in anti-oxidative and anti-inflammatory capacity in both humans and experimental animals [40, 47]. Although a human study reported that low-calorie intake reduces the risk of developing AD [106], inconsistent results were obtained in various AD models and with different types of food restriction paradigms. While some studies found attenuated Aβ deposition [107, 108], others report no effects on Aβ plaque loads despite improved performances in cognitive tests [44]. Our previous results demonstrating exacerbation of AD-like neurodegeneration and neuroinflammation by EOD feeding suggest that this intervention might be too mild to counteract the fast disease progression observed in this particular model, also known to exhibit spontaneous, mostly nonconvulsive epileptic activity that may interfere with EOD feeding-induced effects and be one of the study limitations. Still, new evidence that four months of food restriction can restrain PV-INs loss prompts further analysis of the potential of dietary interventions to counteract early neurodegenerative changes by modulating neurotrophic signaling and neuronal survival pathways. Negative association of EOD-induced increase in PV-positive neuron density with pathology, and some improvements in behavioral deficits imply an attempt at compensatory reorganization of inhibitory circuits in response to network stress and introduces an important aspect for future investigations, shifting the focus away from a simple narrative of PV interneuron loss under pathological conditions, and towards understanding the possibility of complex, adaptive remodeling attempts that may significantly affect disease progression and therapeutic outcomes.

An additional study limitation is that focusing solely on PV-positive interneurons cannot capture the full complexity of GABAergic pathology, including their region-specific vulnerability. Future comprehensive studies are thus warranted to clarify the specific roles of other critical components of inhibitory circuits implicated in AD pathology, such as interneurons expressing somatostatin, calbindin, and others. This is also particularly important for understanding the long-term translational implications of interventions such as EOD, their interplay with Aβ pathology across diverse neuronal populations, and therapeutic targeting in AD. The potential of EOD feeding to impose additional stress on neural circuits must not be overlooked as well. Thus, a comprehensive perspective, highlighting both therapeutic promise and limitations, is warranted for outlining critical future directions.

## Supplementary Information

Below is the link to the electronic supplementary material.

Supplementary file 1 (DOCX 7.30 MB)

## Data Availability

No datasets were generated or analysed during the current study.
