# Supplementary material for: Every-Other-Day Feeding Prevents the Loss of Parvalbumin-Expressing Neurons in the Cerebral Cortex of Female 5xFAD Mice
Source: Mol Neurobiol. 2025 Nov 28;63(1):195. doi: 10.1007/s12035-025-05355-w (PMC12662871; doi:10.1007/s12035-025-05355-w)

Submission ID: 63a91171-60dc-46f5-ac71-03628cbdca53

Title: **Every-other-day feeding prevents the loss of parvalbumin-expressing neurons in the cerebral cortex of female 5xFAD mice**

Authors: Jelena Ciric, Milka Perovic, Nikola Milovanovic, Suryanarayana Polaka, Irena Jovanovic Macura, Natasa Nestorovic, Vesna Tesic

Western blot membranes containing 5, 10, or 15 μg of total protein were successively cut and incubated with specific antibodies. Below are representative blue X-ray films capturing the chemiluminescent signals from repeated experiments (technical replicates). Signals following appropriate housekeeping genes immunoblotting, used as loading controls, are displayed on the right. To minimize positional bias, such as edge effects, and to ensure randomization for more reliable comparisons, the sample order was varied across successive membranes.

**Figure 3A**

Membrane sections between 25 and 70 kDa were blotted with anti-proBDNF antibody, following β-Actin (loading control) and pCaMKII immunostaining and additional cutting at 55 kDa (appropriate band is indicated by an arrow).


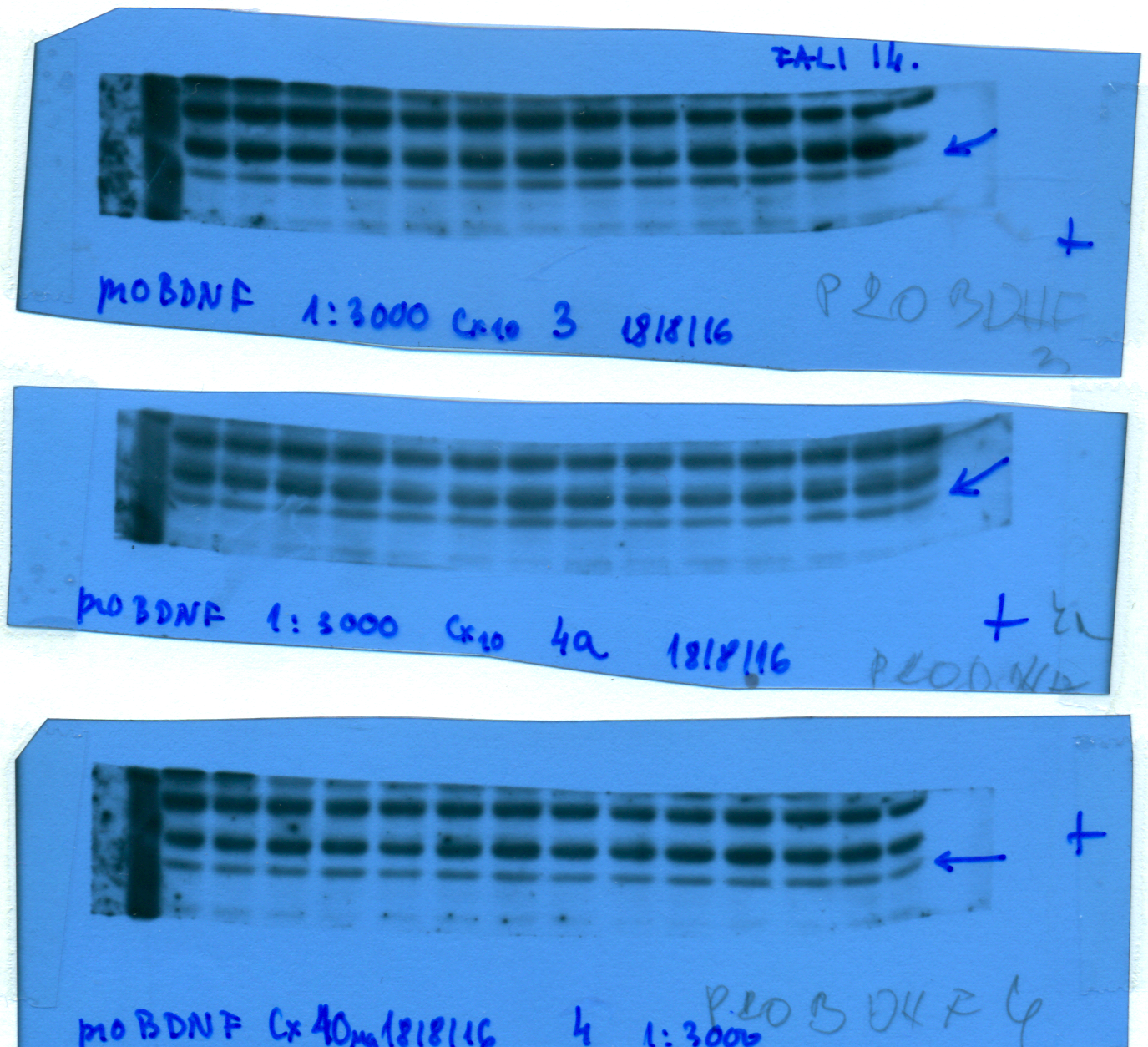

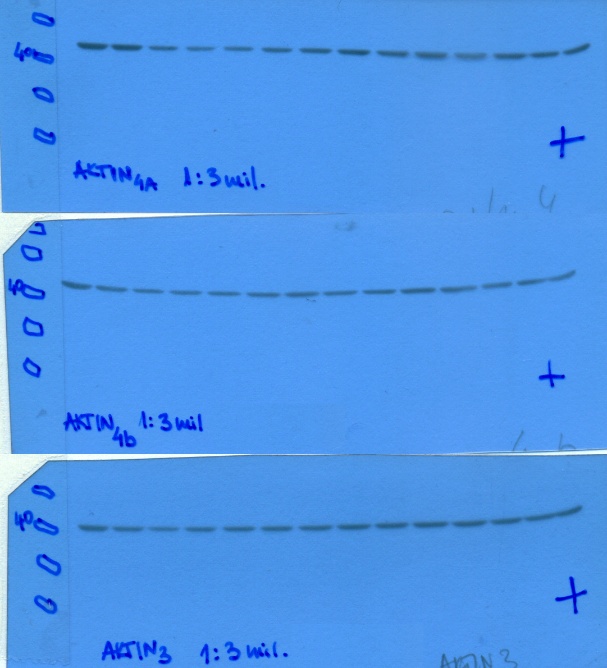


**Figure 3B**

Membrane sections below 25 kDa were

blotted with anti-**BDNF** antibody (14 kDa) Housekeeping gene - **β-Actin** (43 kDa)


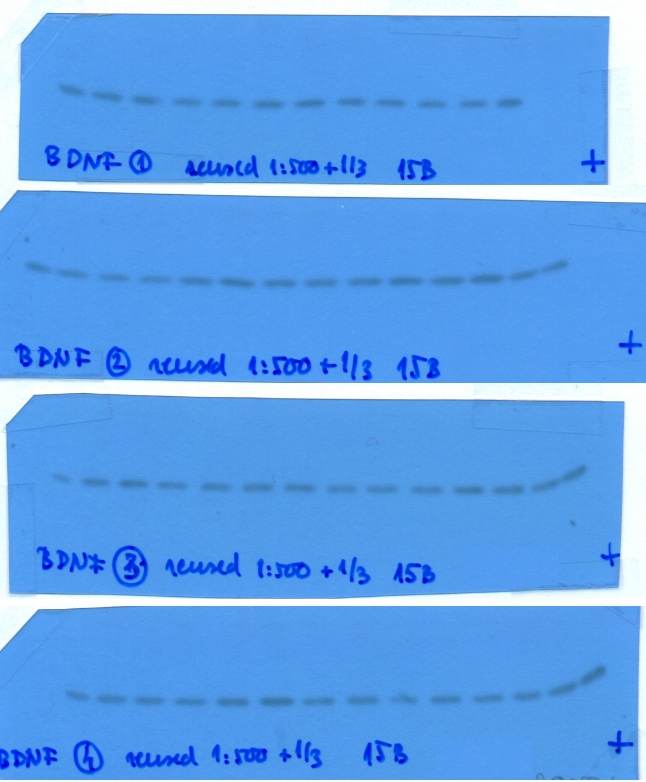

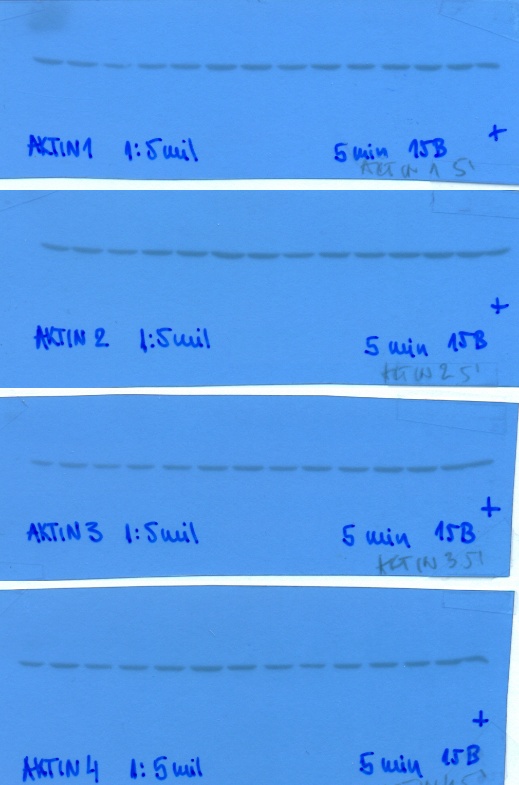


**Figure 4**

For TrkB/pTrkB detection, the membranes were cut to >70 kDa and

**Figure 4A**

Blotted with anti-**TrkB** antibody (130 kDa) Housekeeping gene - **GAPDH** (36 kDa)


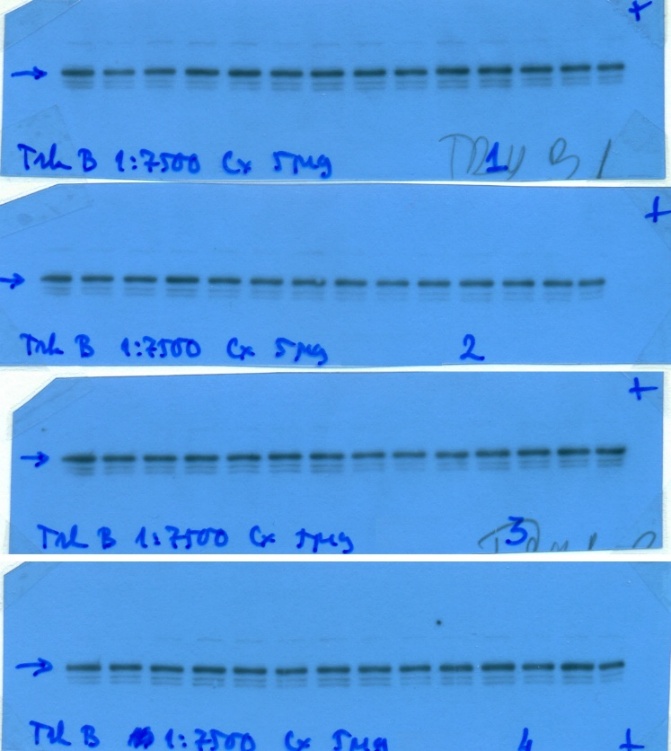

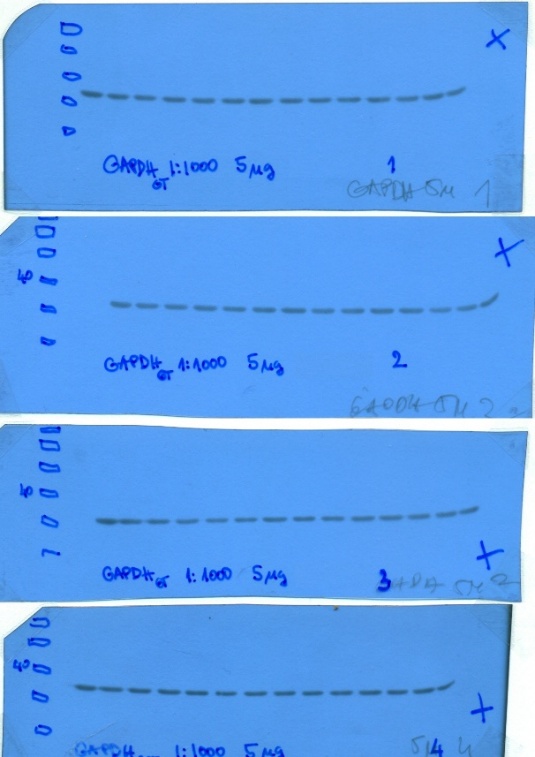


**Figure 4B**

Blotted with anti-p**TrkB** antibody (130 kDa) Housekeeping gene - **β-Actin** (43 kDa)


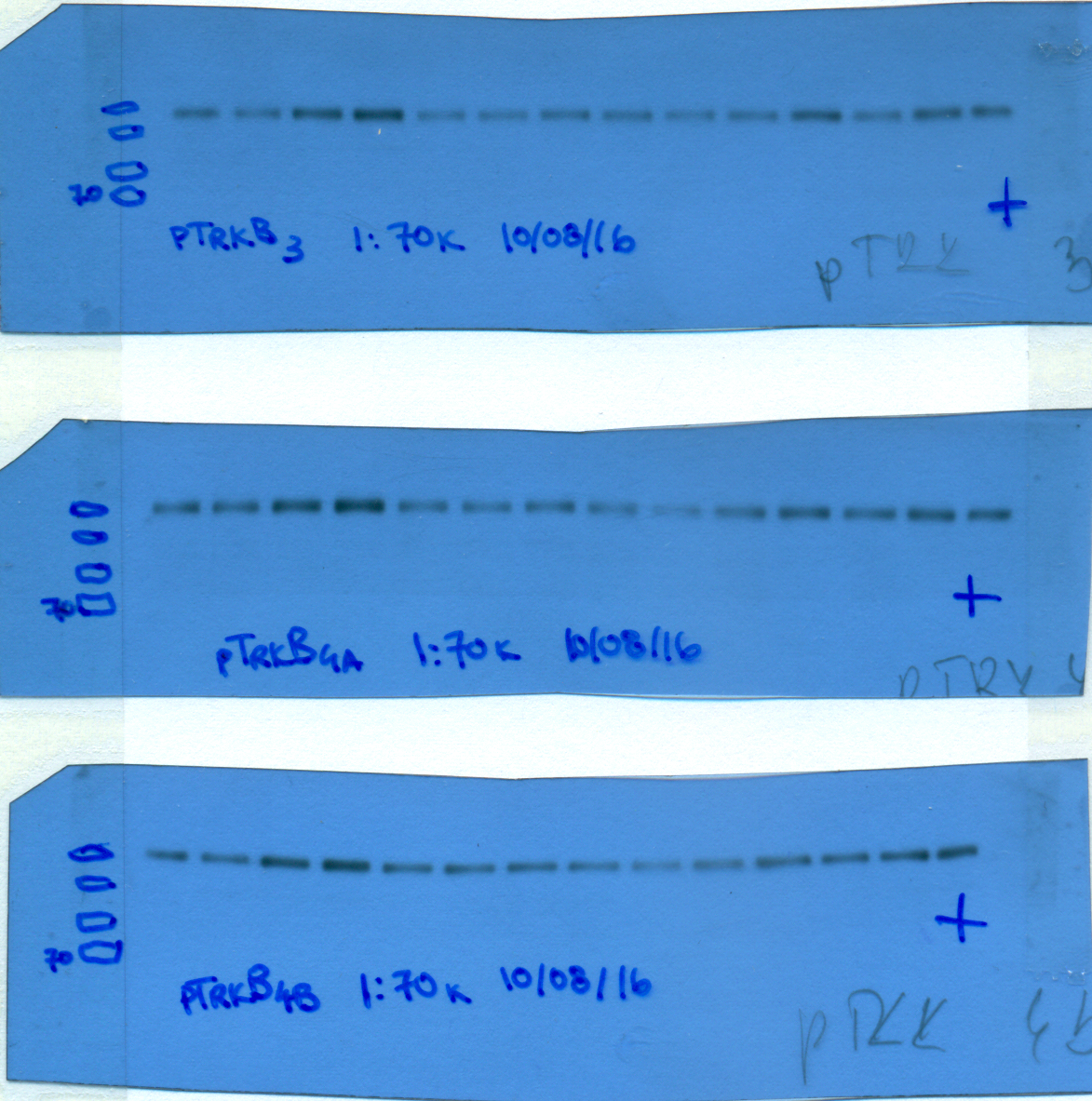

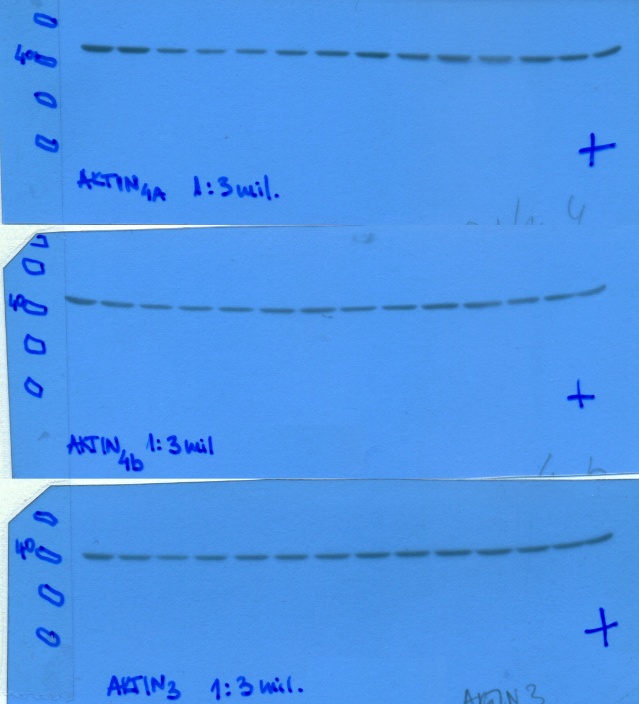


**Figure 5A**

pCaMKII signals (50 kDa; indicated by an arrow) were obtained on the sections between 25 and 70 kDa following the β-Actin (loading control) immunoblotting

Housekeeping gene - **β-Actin** (43 kDa)


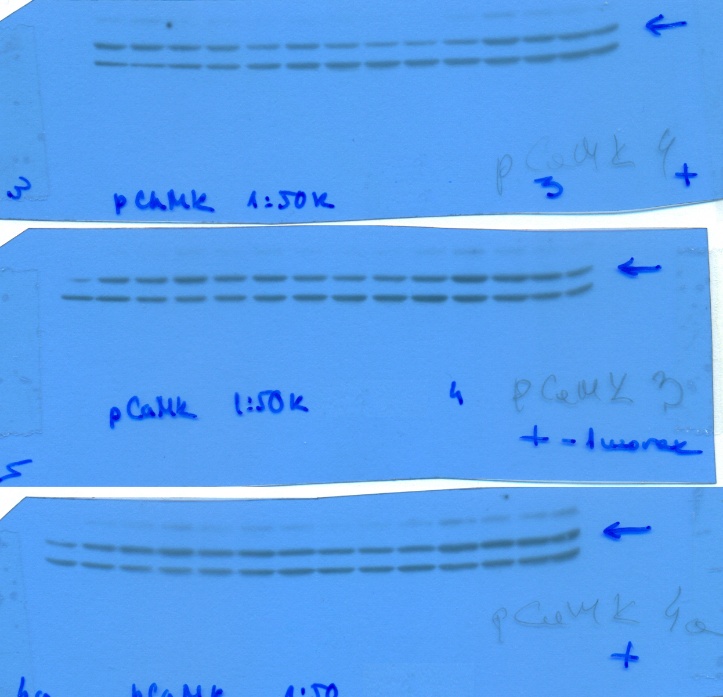

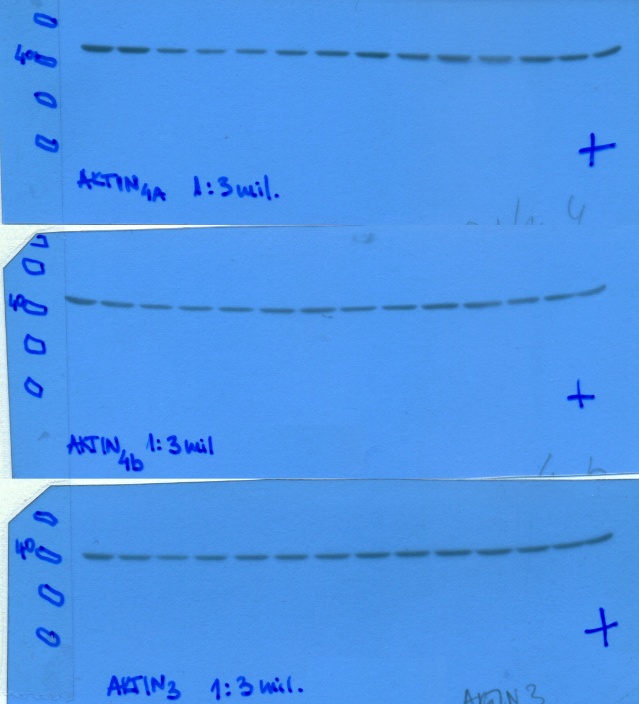


**Figure 5B**

The membrane sections >70 kDa were

Blotted with anti-CBP antibody Housekeeping gene - **GAPDH** (36 kDa)


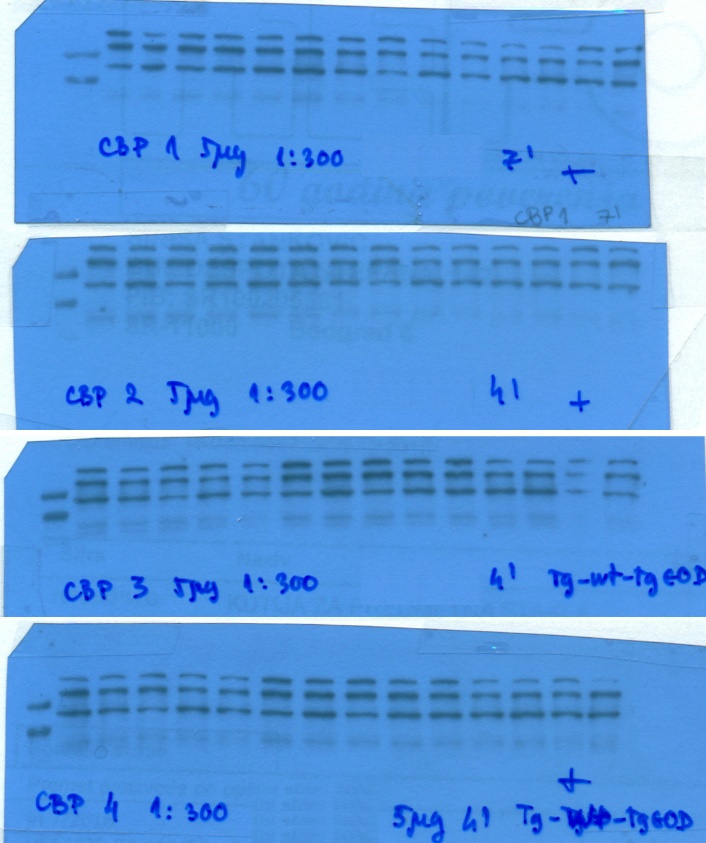

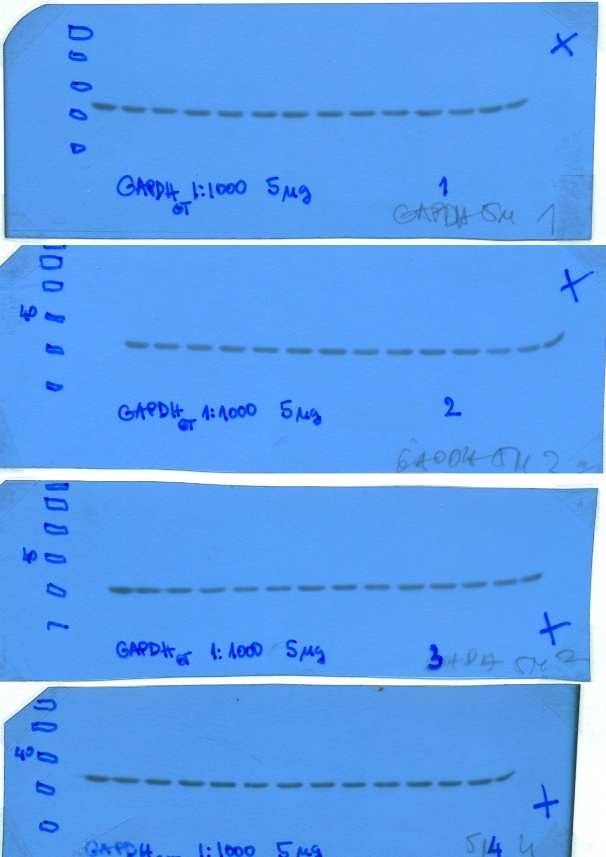

Supplement: Supplementary file 1 — (DOCX 7.30 MB) [file 12035_2025_5355_MOESM1_ESM.docx]
